# CFTR reduces the proliferation of lung adenocarcinoma and is a strong predictor of survival in both smokers and non-smokers

**DOI:** 10.1007/s00432-022-04106-x

**Published:** 2022-06-17

**Authors:** Qingyang Xiao, Stefania Koutsilieri, Despoina-Christina Sismanoglou, Volker M. Lauschke

**Affiliations:** 1 Department of Physiology and Pharmacology, Karolinska Institutet, 171 77 Stockholm, Sweden; 2 Department of Pharmacy, University of Patras School of Health Sciences, Patras, Greece; 3 Dr Margarete Fischer-Bosch Institute of Clinical Pharmacology, Stuttgart, Germany; 4 University of Tuebingen, Tuebingen, Germany

**Keywords:** *ABC* transporters, Prognostic biomarkers, Drug resistance, Gene expression, Ivacaftor, Expression signature

## Abstract

**Background:**

One of the main hurdles of oncological therapy is the development of drug resistance. The *ABC* transporter gene family contributes majorly to cancer chemoresistance. However, effects of somatic expression of most *ABC* transporters on cancer outcomes remain largely unclear.

**Methods:**

We systematically analyzed expression signatures of all 48 human *ABC* transporters in samples from 8562 patients across 14 different cancer types. The association between *CFTR* (*ABCC7*) expression and outcomes was analyzed experimentally using knock-downs and pharmacological CFTR stimulation.

**Results:**

Across 720 analyzed clinical associations with patient outcomes, 363 were nominally significant of which 29 remained significant after stringent Bonferroni correction. Among those were various previously known associations, as well as a multitude of novel factors that correlated with poor prognosis or predicted improved outcomes. The association between low *CFTR* levels and reduced survival in lung adenocarcinoma was confirmed in two independent cohorts of 246 patients with a history of smoking (logrank *P* = 0.0021, hazard ratio [HR], 0.49) and 143 never-smokers (logrank *P* = 0.0023, HR 0.31). Further in vitro experiments using naturally *CFTR* expressing lung adenocarcinoma cells showed that treatment with CFTR potentiators significantly reduced proliferation at therapeutically relevant concentrations.

**Conclusions:**

These results suggest that CFTR acts as a pharmacologically activatable tumor suppressor and constitutes a promising target for adjuvant therapy in lung adenocarcinoma.

**Supplementary Information:**

The online version contains supplementary material available at 10.1007/s00432-022-04106-x.

## Introduction

ATP-binding cassette (ABC) transporters are membrane proteins that catalyze the translocation of a wide range of endogenous substrates, such as hormones, carbohydrates, various inorganic anions and antioxidants, as well as a large number of drugs. *ABC* transporter-mediated efflux is particularly important in oncology where it results in increased extrusion of a multitude of structurally diverse chemotherapeutic substrates, including anthracyclines, camptothecins, vinca alkaloids, taxanes and methotrexate, and constitutes a major hallmark of multidrug resistance (Xiao et al. 2021).

Increased drug efflux can be due to variation in *ABC* transporter genes resulting in increased transporter activity. Examples are variants in *ABCB1* that can predict toxicity and response to chemotherapy in breast cancer (Xiao et al. 2020) and *ABCG2* and *ABCC10* variability that associates with tyrosine kinase inhibitor and taxane toxicity in non-small cell lung cancer (Noguchi et al. 2014; Sone et al. 2019). However, the predictive accuracy of *ABC* transporter germline variants remains low, and they are thus currently not suitable to guide cancer therapy selection and posology. In contrast to genetic variability, the expression levels of *ABC* transporters are more accurate in predicting chemotherapy resistance and can serve as robust biomarkers for remission and survival across various cancers, including hematological cancers, as well as various solid tumors, such as melanoma, colorectal cancer, ovarian cancer, Ewing sarcoma and breast cancer (Pasello et al. 2019; Fletcher et al. 2010). While the mechanisms underlying *ABC* transporter overexpression are not fully understood, causes are likely gene induction via trans acting mechanisms or the selection of intrinsically overexpressing subclones (Theile and Wizgall 2021).

While numerous studies demonstrated associations between *ABC* transporter expression, chemotherapy resistance and patient prognosis, most research has focused on *ABCB1* (encoding P-gp/MDR1), *ABCG2* (encoding BCRP) and *ABCC1* (encoding MRP1), and relatively little is known about how expression levels of other *ABC* transporters impact multidrug resistance, remission, and survival. Importantly, the rapidly increasing wealth of large-scale cancer transcriptomic data now enables for the first time the systematic evaluation of *ABC* transporter expression signatures across multiple cancer types.

In this study, we comprehensively analyzed associations between expression of all 48 members of the human *ABC* transporter supergene family and clinical outcomes across 14 cancer types by parsing sequencing and microarray data of a total of 8,562 patients. Across 720 analyzed clinical associations, we identified 29 links between *ABC* expression and overall survival that were significant after stringent Bonferroni multiple testing correction. Among these, expression levels of *CFTR* (*ABCC7*) showed a significant inverse correlation with overall survival in lung adenocarcinoma (logrank *P* = 3*10^–5^; hazard ratio [HR], 0.54; 95% confidence interval [CI], 0.41–0.73), an association that was confirmed in independent lung adenocarcinoma validation cohorts of smokers (logrank *P* = 0.0021; HR, 0.49; CI, 0.3 to 0.78) and never-smokers (logrank *P* = 0.0023; HR, 0.31; CI, 0.14 to 0.68). Importantly, in vitro experiments using *CFTR* expressing lung adenocarcinoma cells showed that exposure to therapeutic levels of the CFTR potentiator ivacaftor, clinically approved for the treatment of cystic fibrosis, significantly reduced proliferation and showed additive effects with anthracyclines. Combined, the presented study identified *CFTR* expression as a prognostic marker for survival in lung adenocarcinoma and suggests that stimulation of CFTR function using safe concentrations of ivacaftor might provide a novel strategy to reduce tumor cell proliferation.

## Methods

### Data sources

ABC transporter expression data and the corresponding patient survival data were obtained from the cancer genome atlas program (TCGA) repository, the cancer biomedical informatics grid and the gene expression omnibus and analyzed as previously (Nagy et al. 2021; Győrffy 2021). The discovery cohort consisted of RNA sequencing data of 530 clear cell renal cell carcinoma (ccRCC), 288 papillary renal cell carcinoma (PRCC), 177 pancreatic ductal adenocarcinoma (PDAC), 371 hepatocellular carcinoma (HCC), 304 cervical squamous cell carcinoma (CSCC), 375 gastric adenocarcinoma, 501 lung squamous cell carcinoma (LSCC), 504 lung adenocarcinoma, 374 ovarian cancer, 405 bladder carcinoma, 500 head-and-neck squamous cell carcinoma (HNSCC), 259 sarcoma, 543 endometrial carcinoma, as well as microarray data of 1,496 and 1,935 breast cancer (BRCA) samples from patients who received or did not receive endocrine therapy, respectively. Survival analysis of samples with RNA sequencing information was performed as previously reported (Nagy et al. 2021). Overall survival was used as endpoint for all cancer types, except for breast cancer, for which relapse-free survival was used concordant with current recommendations (Liu et al. 2018). In addition, microarray expression data from 1,656 ovarian cancers and 719 lung adenocarcinoma samples were obtained and analyzed as previously reported for independent validations (Győrffy et al. 2012, 2013). Logrank *P* values were subjected to stringent Bonferroni multiple testing correction accounting for 720 tests (48 *ABC* transporter genes and 14 cancer types of which breast cancer samples were considered as two cohorts, one receiving endocrine therapy and one without, resulting in 48*15 = 720 tests). All analyzed data were freely available to the public for deidentified analysis and separate IRB approval was not required. *P* < 0.05 was considered as statistically significant.

### Cell culture

Calu-3 HBT-55 cells were grown in MEM Eagle medium (Sigma) supplemented with 10% heat inactivated fetal bovine serum (FBS; Gibco), non-essential amino acids (Sigma), 2 mM l-glutamine (Sigma), 1 mM sodium pyruvate (Sigma) and penicillin–streptomycin (Cytiva). This cell line was chosen over the commonly used A549 cells as the latter do not express CFTR. Cells were propagated at 37 °C in 5%CO_2_ in a humidified incubator for five passages before transfection. Cells (96,000 cells/ml) were transfected in suspension with 10 nM endoribonuclease prepared siRNA consisting of a heterogeneous mixture of different CFTR targeting siRNAs target (EHU020491, Sigma) using lipofectamine RNAiMAX (Invitrogen) in antibiotics-free medium. Subsequently, the transfected cells were seeded in 24-well plates at a density of 48,000 cells per well (20% confluency). Calu-3 cells were tested negative for mycoplasma contamination.

### Gene expression analysis

Total RNA was extracted using the Quick-RNA MiniPrep kit (Zymo Reseach) and 50 ng were reverse transcribed using the SuperScript III Reverse Transcriptase kit (Invitrogen). *CFTR* expression levels were quantified on a 7500 Fast Real-Time PCR System (Applied Biosystems) using the gene specific TaqMan probe (Hs00357011_m1, ThermoFisher) and normalized to *TBP* (Hs00427620_m1). Relative expression and fold-changes were calculated using the ΔΔC_T_ method.

### Western blot

Cell extracts for both knock-downs and untreated controls were prepared in RIPA buffer (Thermo) supplemented with complete protease inhibitor tablets (Roche). The total protein concentration was determined via the Lowry method (BioRad) and 10ug of total protein were separated by 10% SDS–polyacrylamide gel (Mini PROTEAN TGX, BioRad) electrophoresis. The membrane was blocked with 5% milk protein (Cell Signaling) in TBST (20 mM Tris, pH = 7,5, 150 mM NaCl, 0,1% Tween 20) and probed overnight at 4 °C with mouse monoclonal anti-CFTR antibody (sc-376683, Santa Cruz) and β-actin (A5441, Sigma-Aldrich). As secondary antibody, we used horseradish peroxidase-conjugated donkey anti-mouse IgG (A16017, Invitrogen).

### Proliferation assay

48 h after transfection, cells were exposed to the CFTR potentiator ivacaftor (Sigma) and/or the anthracycline doxorubicin as indicated. The final concentration of DMSO in all treatment conditions and the control group was 0.3% v/v. Following exposure, cells were immediately placed in an incubator outfitted with an IncuCyte S3 (ESSEN Bioscience) microscope and phase-contrast images of 16 fields per well were automatically acquired every 4 h over an 80 h time course and confluence was determined automatically using the integrated software (S3 2019B Rev3). All conditions were performed in biological triplicates. Cell numbers were normalized to the first scan of the well right after the exposure. Statistical significance was determined using heteroscedastic two-tailed *t* tests or *F* tests as indicated. *P* < 0.05 was considered significant.

## Results

### Systematic analysis of ABC transporter signatures across 14 cancer types

**Fig. 1:**
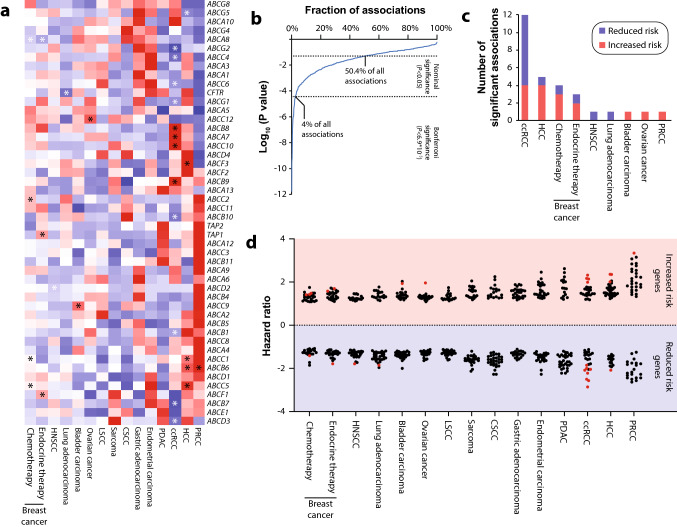
The landscape of somatic *ABC* transporter expression alterations across 14 cancer types. **a** Mean-centered heatmap of the hazard ratios for expression of all 48 human *ABC* transporter genes across 14 different cancers. Cancer types and genes are sorted using unsupervised hierarchical clustering. Red and blue cell shades indicate reduced and increased survival in patients with elevated somatic expression. Asterisks indicate associations (*n* = 29) that were significant after Bonferroni correction. **b** Distribution of test significances. 50.4% of all associations were nominally significant (logrank *P* < 0.05) with 4% remaining significant after correction for multiple testing (logrank *P* < 6.9*10^–5^). **c** Bar plot showing the number of significant associations after multiple testing correction per cancer type. **d** Dot plot showing the hazard ratio distributions for the different cancer types. Significant associations after multiple testing correction are shown as red dots. Note that ABC expression effect sizes differ considerably between cancer types. *BRCA*   breast cancer, *ccRCC* clear cell renal cell carcinoma, *CSCC* cervical squamous cell carcinoma, *HCC* hepatocellular carcinoma, *HNSCC* head-and-neck squamous cell carcinoma, *LSCC* lung squamous cell carcinoma, *PDAC* pancreatic ductal adenocarcinoma, *PRCC* papillary renal cell carcinoma

**Fig. 2:**
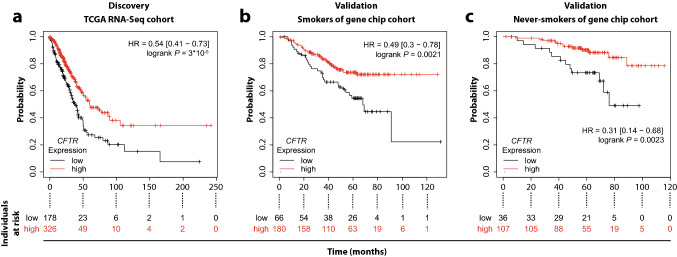
Association of *CFTR* with increased overall survival of lung adenocarcinoma patients across three independent cohorts. Kaplan–Meier plots of patient survival stratified by *CFTR* expression in the TCGA discovery cohort (**a**; *n* = 504 patients), as well as in two independent validation cohorts of smokers (**b**; *n* = 246 patients) and never-smokers (**c**; *n* = 143 patients) from the GeneChip data set

We first parsed the expression of all 48 human *ABC* transporter genes across 14 different types of solid cancers using comprehensive transcriptomic data of 8,562 matched tumor and peritumoral tissue samples (Fig. 1a). Specifically for breast cancer, we stratified patients into whether or not they received endocrine therapy. When correlating tumor expression status with survival, we identified a total of 363 nominally significant associations (50.4% of all 720 tests) of which 29 remained significant after stringent Bonferroni multiple testing correction (resulting Bonferroni family-wise error threshold, 6.9*10^–5^; Fig. 2b). The largest numbers of significant ABC transporter associations were identified for ccRCC (*n* = 12), HCC (*n* = 5) and BRCA (*n* = 4 for chemotherapy and *n* = 3 for endocrine cohort), whereas only a single significant correlation was found for lung adenocarcinoma, PRCC, HNSCC, bladder carcinoma and ovarian cancer (Fig. 1c). No significant associations of *ABC* transporter expression with survival were found for endometrial cancer, sarcoma, LSCC, CSCC and gastric adenocarcinoma. Notably, the magnitude of associations between *ABC* expression and patient outcomes differed considerably across cancer types (Fig. 1d). Hazard ratios were overall lowest for breast cancer and HNSCC, whereas the largest effect sizes were observed for renal cancers (PRCC and ccRCC) and HCC.

Drug efflux catalyzed by the multidrug resistance transporters MDR1 and MRP1 constitutes an essential mechanism of cancer chemoresistance. Previous research revealed associations of *ABCB1* transcript levels with poor outcomes in ovarian cancer (Johnatty et al. 2013; Sun et al. 2016), gastric cancer (Oliveira et al. 2014) and HCC (Fan et al. 2016), whereas inverse trends have been reported for HNSCC (Warta et al. 2014) and ccRCC (Reustle et al. 2018). In agreement with these findings, we observed protective effects of *ABCB1* expression on survival in ccRCC (logrank *P* = 1.4*10^–6^, HR 0.49, CI 0.36–0.66) and HNSCC (logrank *P* = 9.6*10^–5^, HR 0.59, CI 0.43–0.74; Table 1). Furthermore, inverse trends were observed for ovarian cancer (logrank *P* = 0.052, HR 1.32, CI 1.0–1.74) and HCC (logrank *P* = 0.002, HR 1.75, CI 1.22–2.52) that were however not significant after correction for multiple testing. Our results also corroborated (HCC: logrank *P* = 5.4*10^–5^, HR 2.01, CI 1.42–2.84; BRCA: logrank *P* = 2.2*10^–5^, HR 1.43, CI 1.21–1.68) previous observations that increased expression of the multidrug resistance transporter MRP1 associated with poor prognosis in HCC (Vander Borght et al. 2008) and BRCA patients undergoing chemotherapy (Filipits et al. 2005).

**Table 1:** Significant associations between tumoral *ABC* transporter expression and patient outcomes. BRCA_chemo_ = breast cancer patients who received only chemotherapy; BRCA_endo_ = breast cancer patients who received endocrine therapy; ccRCC = clear cell renal cell carcinoma; HCC = hepatocellular carcinoma; HNSCC = head-and-neck squamous cell carcinoma; HR = hazard ratio; PRCC = papillary renal cell carcinoma

Cancer type	ABC transporter	Logrank *P*-values		HR	HR 95% confidence interval
		Nominal	Bonferroni corrected		
*Increased risk associations*					
PRCC	*ABCB6*	2.8*10^–5^	0.02	3.34	1.84–6.06
HCC	*ABCB6*	9.5*10^–7^	6.8*10^–4^	2.36	1.66–3.36
HCC	*ABCC5*	7.1*10^–7^	5.1*10^–4^	2.35	1.66–3.32
ccRCC	*ABCC10*	2.9*10^–8^	2.1*10^–5^	2.32	1.71–3.14
ccRCC	*ABCA7*	4.8*10^–6^	0.003	2.17	1.54–3.04
ccRCC	*ABCB9*	4.2*10^–7^	3*10^–4^	2.14	1.58–2.88
HCC	*ABCF3*	4.4*10^–5^	0.032	2.05	1.44–2.92
HCC	*ABCC1*	5.4*10^–5^	0.039	2.01	1.42–2.84
ccRCC	*ABCB8*	1.2*10^–5^	0.009	1.99	1.45–2.73
Ovarian cancer	*ABCC12*	7.4*10^–6^	0.005	1.96	1.45–2.65
Bladder carcinoma	*ABCC9*	4.8*10^–5^	0.035	1.93	1.4–2.66
BRCA_endo_	*ABCF1*	9.6*10^–7^	6.9*10^–4^	1.7	1.37–2.11
BRCA_endo_	*TAP1*	4.5*10^–5^	0.032	1.58	1.27–1.97
BRCA_chemo_	*ABCC2*	3.1*10^–6^	0.002	1.48	1.25–1.75
BRCA_chemo_	*ABCC1*	2.2*10^–5^	0.016	1.43	1.21–1.68
BRCA_chemo_	*ABCC5*	4.2*10^–5^	0.03	1.41	1.19–1.66
*Reduced risk associations*					
ccRCC	*ABCG2*	1.8*10^–12^	1.3*10^–9^	0.35	0.26–0.48
ccRCC	*ABCB7*	6.4*10^–10^	4.6*10^–7^	0.39	0.29–0.54
ccRCC	*ABCC4*	3.6*10^–10^	2.6*10^–7^	0.4	0.29–0.53
ccRCC	*ABCD3*	1.4*10^–7^	1*10^–4^	0.45	0.33–0.61
ccRCC	*ABCB10*	1.1*10^–6^	7.9*10^–4^	0.48	0.36–0.65
HCC	*ABCG5*	2.6*10^–5^	0.019	0.48	0.34–0.68
ccRCC	*ABCB1*	1.4*10^–6^	0.001	0.49	0.36–0.66
ccRCC	*ABCG1*	5.4*10^–6^	0.004	0.5	0.37–0.68
Lung adenocarcinoma	*CFTR*	3*10^–5^	0.022	0.54	0.41–0.73
ccRCC	*ABCC6*	5.1*10^–5^	0.037	0.54	0.4–0.73
HNSCC	*ABCD2*	2.2*10^–5^	0.016	0.56	0.43–0.74
BRCA_endo_	*ABCA8*	1.9*10^–6^	0.001	0.56	0.44–0.71
BRCA_chemo_	*ABCA8*	2.6*10^–5^	0.019	0.71	0.61–0.83

Besides these confirmations of known multidrug resistance transporters, multiple significant associations with ATP transporters were discovered that were not previously implicated in drug resistance (Table 1). Among the risk factors, high expression of *ABCB8* (logrank *P* = 1.2*10^–5^, HR 1.99, CI 1.45–2.73) and *ABCB9* (logrank *P* = 4.2*10^–7^, HR 2.14, CI 1.58–2.88) were strong predictors of poor prognosis in ccRCC, while *ABCB6* correlated with reduced survival in HCC (logrank *P* = 9.5*10^–7^, HR 2.36, CI 1.66–3.36) and PRCC (logrank *P* = 2.8*10^–5^, HR 3.34, CI 1.84–6.06). Furthermore, expression of the orphan transporters *ABCF3* and *ABCF1* was increased with worse outcomes in HCC (logrank *P* = 4.4*10^–5^, HR 2.05, CI 1.44–2.92) and in BRCA patients undergoing endocrine therapy (logrank *P* = 9.6*10^–7^, HR 1.7, CI 1.37–2.11).

Inversely, expression levels of the peroxisomal fatty acid transporters *ABCD2* (logrank *P* = 2.2*10^–5^, HR 0.56, CI 0.43–0.74) and *ABCD3* (logrank *P* = 1.4*10^–7^, HR 0.45, CI 0.33–0.61), as well as the mitochondrial transporters *ABCB7* (logrank *P* = 6.4*10^–10^, HR 0.39, CI 0.29–0.54) and *ABCB10* (logrank *P* = 1.1*10^–6^, HR 0.48, CI 0.36–0.65) were associated with significantly better outcomes in ccRCC and HNSCC. Further protective associations included the expression of the *ABCG* subfamily member *ABCG1* (logrank *P* = 5.4*10^–6^, HR 0.5, CI 0.37–0.68), *ABCG2* (logrank *P* = 1.8*10^–12^, HR 0.35, CI 0.26–0.48) and *ABCG5* (logrank *P* = 2.6*10^–5^, HR 0.48, CI 0.34–0.68) in ccRCC and HCC, as well as correlations of *CFTR* levels with improved outcomes in lung adenocarcinoma (logrank *P* = 3*10^–5^, HR 0.54 CI 0.41–0.73).

### Validation of selected candidate associations in independent cohorts

To further increase the confidence in the reported associations, we conducted non-parametric survival analysis based on the Kaplan–Meier estimator and aimed for validation of selected associations in independent cohorts. Specifically, we focused on the associations in ovarian cancer and lung adenocarcinoma as the two cancer types for which we had access to sufficiently large validation cohorts. For ovarian cancer, we found a borderline significant association between *ABCC12* expression and overall survival for grade 3 tumors (logrank *P* = 0.048; HR, 1.36; CI 1 to 1.85); however, this association was lost when all cancer grades were considered (logrank *P* = 0.16; Supplementary Fig. 1).

In contrast, the association of increased *CFTR* expression with improved outcomes in lung adenocarcinoma was confirmed in two independent cohorts (Fig. 2). First, we validated the finding in 246 patients with a history of smoking and found a highly significant association with an almost identical hazard ratio compared to the TCGA RNA-Seq data (logrank *P* = 0.0021; HR, 0.49; CI 0.3 to 0.78 vs. HR, 0.54; CI, 0.41 to 0.73 for TCGA). Furthermore, we observed a further significant correlation of almost identical magnitude in an additional cohort of 143 never-smokers (logrank *P* = 0.0023; HR, 0.31; CI 0.14 to 0.68).

### Pharmacological stimulation of CFTR function reduces lung adenocarcinoma cell proliferation

**Fig. 3:**
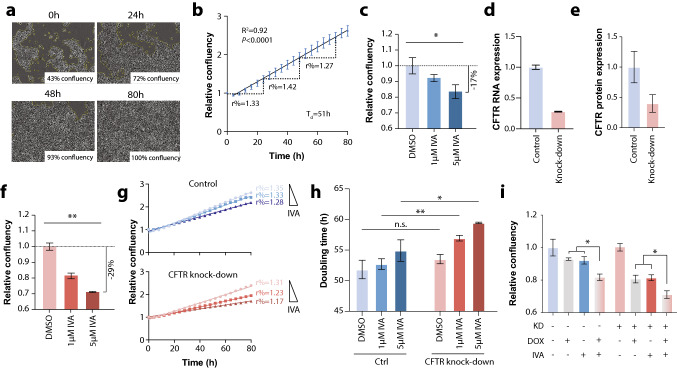
CFTR activation reduces the proliferation of lung adenocarcinoma cells in vitro. **a** Time-lapse brightfield images of Calu-3 cells at selected time points throughout the experimental timeframe of 80 h. Images show the same representative field of view. **b** The relative confluency of Calu-3 cells was determined automatically every 4 h using in three independent samples using nine fields of view each. Note, that cell proliferation in the studied time frame is quasi-linear with a coefficient of determination (*R*^2^) of 0.92. Cellular growth rates (*r*%) are indicated for three intervals (0–24 h, 24–48 h and 48–72 h), resulting in an overall doubling time (*T*_d_) of 51 h. **c** Bar plot showing the reduction in lung adenocarcinoma cell confluency after 80 h upon treatment with ivacaftor compared to DMSO control. Expression levels of CFTR mRNA **d** and *CFTR* protein **e** upon siRNA-mediated knock-down. **f** Lung adenocarcinoma cell confluency after 80 h upon treatment with ivacaftor in *CFTR* knock-down cells. **g** Proliferation curves of control and *CFTR* knock-down cells upon treatment with vehicle (DMSO), 1 and 5 µM ivacaftor. Note the dose-dependent decrease in growth rates. **h** Quantification of cellular doubling times. Ivacaftor dose-dependently increases doubling times in both control and CFTR knock-down cells; however, the magnitude of this effect is significantly higher in the latter. **i** Therapeutic concentrations of doxorubicin (10 nM) and ivacaftor (1 µM) show additive effects in reducing cell numbers. Error bars indicate SEM. * and ** indicate *P* < 0.05 and *P* < 0.01, respectively. *n.s.* not significant (*P* > 0.05). *IVA* ivacaftor, *DOX* doxorubicin; *KD* CFTR knock-down

The clinical association of CFTR expression with lung cancer patient prognosis was further investigated in vitro. Notably, because the most commonly used and highly proliferative lung adenocarcinoma cell line A549 does not express CFTR (Hamai et al. 2009), we used Calu-3 cells, which exhibit natural CFTR expression and thus do not require CFTR overexpression, as in previous studies (Hamai et al. 2009; Qian et al. 2021). To monitor proliferation dynamics, we performed time-lapse imaging and found that Calu-3 cells proliferated with a growth rate (*r*%) of 27–42% per 24 h, resulting in a doubling time (*T*_d_) of ∼51 h (Fig. 3a–b).

Next, we assessed whether CFTR activation impacted cell proliferation. To this end, we exposed cells to clinically relevant concentrations of the CFTR potentiator ivacaftor (1–5 µM; therapeutic *c*_max_∼2–3 µM dose) (https://www.ema.europa.eu/en/documents/product-information/kalydeco-epar-product-information_en.pdf; https://www.accessdata.fda.gov/drugsatfda_docs/nda/2012/203188Orig1s000ClinPharmR.pdf). Notably, we observed a significant dose-dependent inhibition of proliferation by 8 and 17% in cells exposed for three days to 1 µM and 5 µM ivacaftor, respectively (*P* = 0.023; Fig. 3c). To test whether effects of CFTR activation differ with CFTR expression, we used knock-downs using antisense oligos. Transfection with anti-CFTR siRNA reduced RNA and protein levels by 72 and 40%, respectively (Fig. 3d–3; Supplementary Fig. 2). Importantly, in cells in which CFTR expression was reduced, the effects of CFTR potentiation were even stronger with proliferation being reduced by up 29% after three days of exposure (Fig. 3f). These endpoint results align with dynamic proliferation measurements showing that daily growth rates in controls were decreased by 7%, corresponding to increases in cell doubling times from 51.8–54.9 h, while growth rates of CFTR knock-down cells were increased by 14%, resulting in doubling times of 59.4 h (Fig. 3g–h).

We furthermore tested whether the cytostatic effects of ivacaftor were synergistic with conventional cytotoxic chemotherapeutics, such as anthracyclines. Notably, when ivacaftor or doxorubicin were given alone at therapeutic concentrations (doxorubicin, 10 nM) (Bramwell et al. 2002), both drugs equally reduced cell numbers after 3 days of exposure (Fig. 3i). However, when both drugs were combined significant additive effects were observed in both control and CFTR knock-down cells, suggesting that CFTR potentiation might be a promising strategy for adjuvant therapy irrespective of tumor CFTR expression levels.

## Discussion

Cancer gene expression patterns have been established as tools to classify tumor subgroups and derive patient prognoses (Qian et al. 2021). However, except for the FDA-approved Oncotype DX and MammaPrint tests that constitute integral parts of routine clinical care to guide decision-making regarding the use of adjuvant chemotherapy in early breast cancer (van ’t Veer et al. 2002; Paik et al. 2004; Cardoso, et al. 2016), other cancer expression signatures remain, as of to date, not actionable. Besides actionability, tumor signatures can also reveal mechanisms underlying disease biology, thereby potentially informing drug development and future therapeutic strategies. However, statistical association between gene expression patterns and cancer outcomes cannot be directly used to infer disease-relevant mechanisms without careful validation and/or experimental support (Venet et al. 2011).

To increase the likelihood of finding mechanistically meaningful associations, we here focused on the *ABC* transporter gene family due to their known roles in chemotherapy resistance. Importantly, while poor prognosis is conventionally associated with the overexpression of ABC transporters, we find that of the 29 significant associations between expression and survival, only 16 (55%), mostly with members of the *ABCA*, *ABCB* and *ABCC* subfamilies, indicate that increased expression is associated with worse outcomes. By contrast, increased expression of *ABCD* and *ABCG* genes was overall associated with improved survival. Notably, the cancer types for which the most significant changes in *ABC* expression signatures were detected (ccRCC and HCC), originate from cell types with a rich transporter expression portfolio and major roles in the disposition of endogenous and xenobiotic compounds. As such, associations between reduced transporter expression and poor prognosis might be due to increasing tumor dedifferentiation, possibly downstream of constitutive ERK-NRF2 signaling (Vecchio et al. 2014).

In previous studies, *ABC* expression was shown to be a strong predictor of chemotherapy resistance in acute myeloid leukemia (Marzac et al. 2011; Steinbach et al. 2006) and acute lymphoblastic leukemia patients (Efferth et al. 2006). However, links between *ABC* expression and survival in solid tumors are less established and only few such associations have received experimental support. The association between reduced *CFTR* expression and outcomes in patients with lung adenocarcinoma was among the most significant in our data. *CFTR* encodes a cAMP-activated chloride channel that plays essential roles in ion and water fluxes across epithelial tissues. Homozygous or compound heterozygous reduced function variants in *CFTR* are the known cause of cystic fibrosis and somatic *CFTR* mutations are enriched in non-small cell lung cancer (Govindan et al. 2012). Hypermethylation of *CFTR* resulting in transcriptional deactivation constitutes a common hallmark of NSCLC (Son et al. 2011) and reduced CFTR expression is significantly correlated with advanced disease stage and lymph node metastasis (Li et al. 2015). While these combined genetic and epigenetic findings strongly indicate that CFTR expression levels constitute a strong biomarker for lung cancer outcomes, they do not provide information about whether suppression of CFTR is a consequence of cellular transformation or might act as an upstream driver. Furthermore, these studies did not evaluate whether CFTR might constitute a putative therapeutic target.

We thus conducted in vitro experiments using the CFTR potentiator ivacaftor (Goor et al. 2009). Notably, while ivacaftor is only approved for the treatment of cystic fibrosis patients with specific genetic variations, multiple studies have consistently demonstrated that it also activates the unaltered reference transporter present in the utilized cell line (Eckford et al. 2012; Jih and Hwang 2013; Kim et al. 2018). Our results demonstrate that ivacaftor significantly reduced the proliferation of lung adenocarcinoma cells, corroborating that CFTR activity is mechanistically involved in cancer pathogenesis and acts as a pharmacologically targetable tumor suppressor. Effects of ivacaftor were further amplified upon CFTR knock-downs to levels found in heterozygous cystic fibrosis risk allele carriers (knock-downs resulted in 40% reduced protein levels while heterozygous carriers of the most common cystic fibrosis variant p.Phe508del have around 50% lower CFTR levels at the plasma membrane). Additionally, these findings are consistent with previous reports showing that knock-down of *CFTR* increased malignancy in a mouse xenograft model (Li et al. 2015). Similarly, in vitro exposure of lung cancer cells to nicotine resulted in reduced *CFTR* expression and increased cell migration, thus providing a potential link between smoking and disease aggressiveness (Li et al. 2018). However, the finding that low *CFTR* expression associated with worse clinical outcomes also in never-smokers suggests that potential nicotine-mediated downregulation of *CFTR* does not constitute the only mechanism underlying reduced *CFTR* expression.

In vitro studies in recombinant expression systems indicate that expression of functional *CFTR* suppresses NFκB activity (Vij et al. 2009), which in turn constitutes a key mediator controlling epithelial-to-mesenchymal transition (Ma et al. 2021). While the molecular links between *CFTR* activity and NFκB signaling remain elusive, the inhibition of NFκB activation is likely caused by defective CFTR Cl^−^ channel activity rather than protein misfolding, as the expression of different defective *CFTR* channels results in increased NFκB signaling, irrespective of whether the defect is associated with trafficking or channel function (Weber et al. 2001; Dudez et al. 2008).

Our finding that CFTR is directly involved in lung adenocarcinoma progression receives further support from a recent large Danish register study in which morbidity and mortality of 108,035 individuals genotyped for the *CFTR* reduced function variant p.Phe508del was analyzed during up to 15 years of follow-up (Çolak et al. 2020). Strikingly, the authors found that while heterozygous variant carriers did not have reduced lifespan, they had a 52% higher lung cancer risk, thus strongly suggesting that reduced *CFTR* expression constitutes not only a biomarker for lung cancer prognosis but is also directly involved in disease etiology. Furthermore, a meta-analysis of 1159 NSCLC patients analyzing the association between NFκB expression and overall survival found that NFκB levels constitute a prognostic marker for poor outcomes (Gu et al. 2018), providing further in vivo evidence for the important role of the CFTR- NFκB axis.

In conclusion, our data provide the first comprehensive pan-cancer overview of somatic ABC transporter dysregulation and reveal gene- and cancer type-specific signatures that correlate with patient outcomes. Among those, we validate the association between *CFTR* expression and lung adenocarcinoma in two independent cohorts and demonstrate experimentally that pharmacological stimulation of *CFTR* function at therapeutically relevant concentrations results in a dose-dependent inhibition of lung cancer cell proliferation. In the context of previous results, these data indicate that *CFTR* is directly involved in lung cancer onset and progression and that *CFTR* thus constitutes a promising target for adjuvant therapy by repurposing already approved *CFTR* agonists.

## Supplementary Information

Below is the link to the electronic supplementary material.

Supplementary file1 Supplementary Figure 1: Association of ABCC12 with overall survival of ovarian cancer patients. Kaplan-Meier plots of patient survival stratified by ABCC12 expression in the TCGA discovery cohort (**a**; *n*=373 patients), as well as in the GeneChip validation cohort (**b**, **c**). Note that a borderline significant association was found with stage 3 ovarian cancer (**b**; *n*=392 patients), whereas this correlation was lost when all patients were considered (**c**; *n*=655 patients). (PDF 554 kb)

Supplementary file2 Supplementary Figure 2: Western blot of CFTR knock-down experiments. Western blot membranes are shown that were probed for *CFTR* and β-actin. The quantification of these bands are shown in Figure 3**e**. (PDF 3441 kb)

## Data Availability

All analyzed data are available at The Cancer Genome Atlas Program (TCGA) repository, the Cancer Biomedical Informatics Grid and the Gene Expression Omnibus.
