# Supplementary figures and images for: CFTR reduces the proliferation of lung adenocarcinoma and is a strong predictor of survival in both smokers and non-smokers
Source: J Cancer Res Clin Oncol. 2022 Jun 17;148(12):3293–302. doi: 10.1007/s00432-022-04106-x (PMC9587080; doi:10.1007/s00432-022-04106-x)

# Supplementary Figure 2

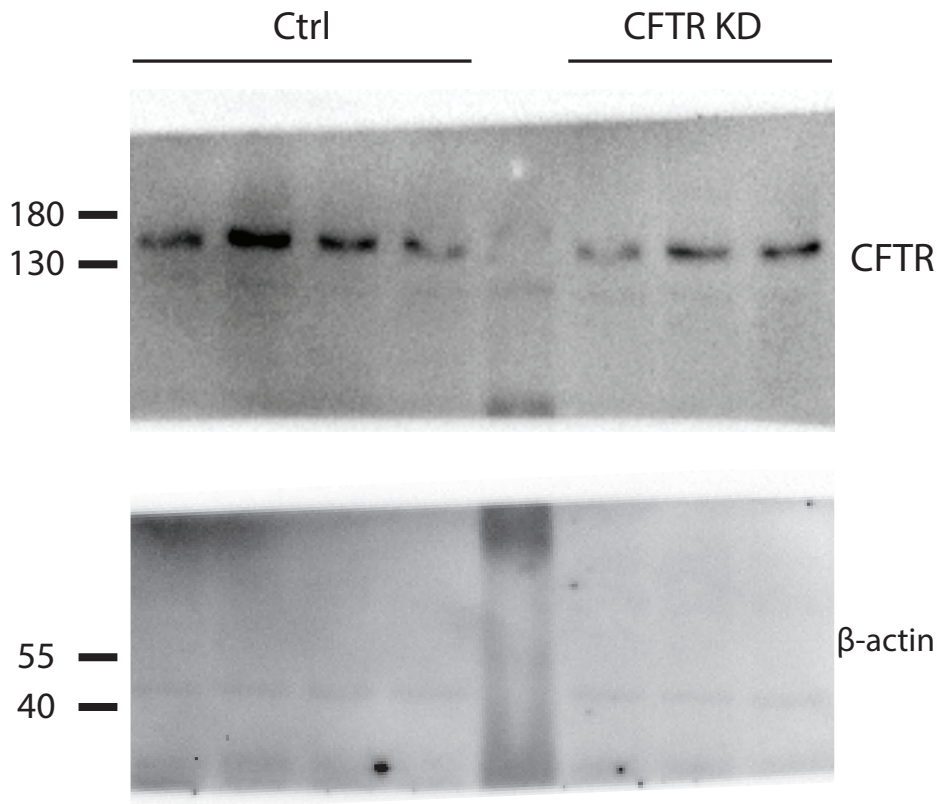

Supplement: Supplementary file 1 — Supplementary file1 Supplementary Figure 1: Association of ABCC12 with overall survival of ovarian cancer patients. Kaplan-Meier plots of patient survival stratified by ABCC12 expression in the TCGA discovery cohort (a; n=373 patients), as well as in the GeneChip validation cohort (b, c). Note that a borderline significant association was found with stage 3 ovarian cancer (b; n=392 patients), whereas this correlation was lost when all patients were considered (c; n=655 patients). (PDF 554 kb) [file 432_2022_4106_MOESM1_ESM.pdf]

# Supplementary Figure 1

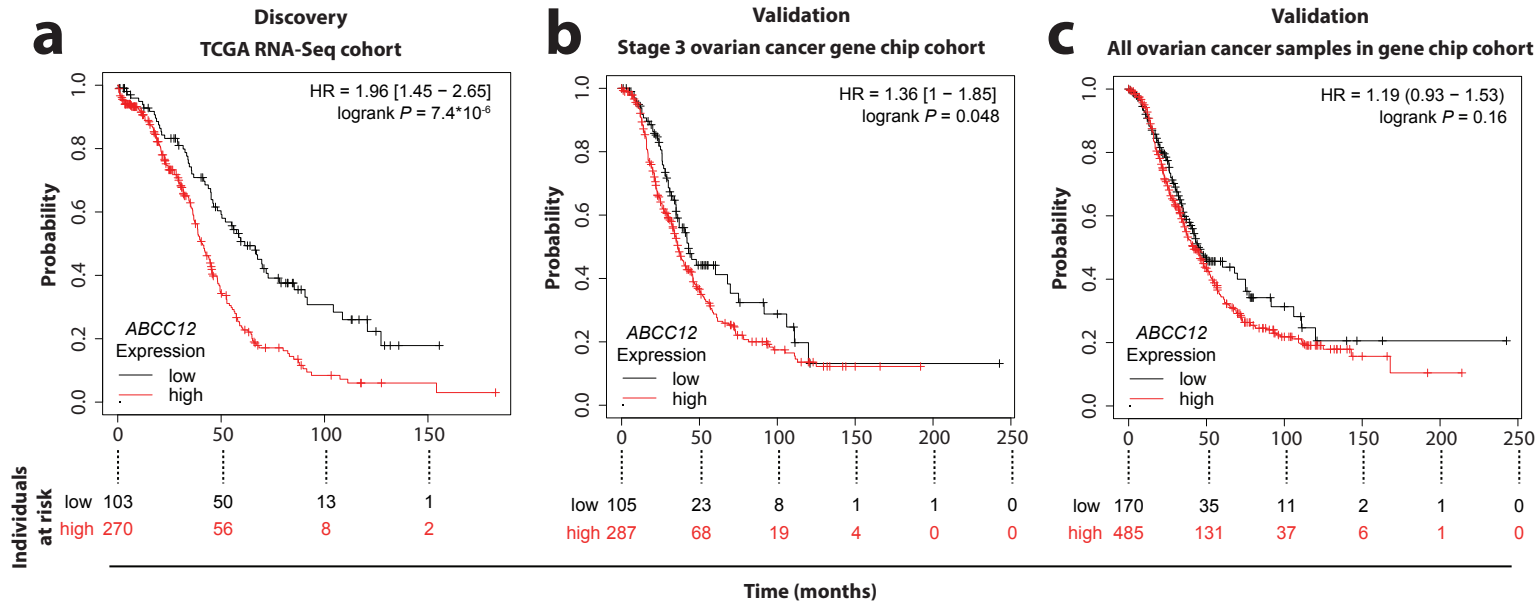

Supplement: Supplementary file 2 — Supplementary file2 Supplementary Figure 2: Western blot of CFTR knock-down experiments. Western blot membranes are shown that were probed for CFTR and β-actin. The quantification of these bands are shown in Figure 3e. (PDF 3441 kb) [file 432_2022_4106_MOESM2_ESM.pdf]
